# Healthcare services utilization among international students in Ankara, Turkey: a cross-sectional study

**DOI:** 10.1186/s12913-021-06301-x

**Published:** 2021-04-07

**Authors:** Abednego Nzyuko Masai, Bahar Güçiz-Doğan, Polet Njeri Ouma, Israel Nyaburi Nyadera, Victor Kipkoech Ruto

**Affiliations:** 1 Department of Public Health, Hacettepe University Faculty of Medicine, Ankara, Turkey; 2 Faculty of Medicine, Hacettepe University, Ankara, Turkey; 3 Department of Political Science and Public Administration, Ankara Yildirim Beyazit University, Ankara, Turkey; 4 Department of Government and Public Administration, University of Macau, Macau, China; 5 Department of International Relations, Ankara University, Ankara, Turkey

## Abstract

**Background:**

While international students form an increasing population of higher education students in Turkey, there is limited empirical evidence about their health services utilization. The study aim was to investigate healthcare access among a group of international students studying in Ankara city and identify potential barriers that affect full healthcare utilization.

**Method:**

A total of 535 international students from 83 countries completed an online-based questionnaire. The survey was conducted from September until October 2020. Variables between groups within the study sample were compared using ANOVA and Chi-square tests (with Fisher’s exact test). Logistic regression analysis was used to evaluate the relationships between variables related to access to health services.

**Results:**

Of the study population, 80.6% accessed the general practitioner (GP), 40% accessed the student health centres, and 11.4% were admitted to the hospital at least once. About 80% of international students reported changing their views to access healthcare more because of the COVID-19 pandemic.

**Conclusion:**

Lack of awareness of healthcare support systems, perceived stigma associated with mental health services, and language barriers were the main barriers affecting healthcare access by international students.

**Implications:**

Study findings indicate the need for education of international students on available healthcare, targeted health promotion, and training of health providers on effective communication.

## Introduction

International students comprise a significant population of higher education students in Turkey. In 2020, the number of international students in Turkey amounted to 185,000, and during the period from 2017 to 2020, the average annual enrollment rate increased up to 15% [1]. As the proportion of international students rise, the awareness and appreciation of their varying needs increases. International students contribute positively to the development of their host countries and their home countries [2]. After completion of their studies, these students contribute to the growth of their home countries by transferring the skills, innovation, and expertise learned abroad.

Starting to live in a new country brings numerous challenges to international students because of culture and language differences [3, 4]. In addition to the pressure associated with the transition to university, international students experience stress related to adapting to a new language, culture, and social way of life [5]. Most international students leave behind their social support network and have to form new ones as they transition to higher education. Lack of social community [6] coupled with the pressure of attaining satisfactory grades [7] results in stress and depression among some international students.

A study by Augoustinos and colleagues (2011) showed that international students reported poorer health and wellbeing [8]. Male students experience more adverse health outcomes and have lower health-seeking behaviors compared to female students [8, 9]. In another study conducted by Skromanis and colleagues (2018), male international students reported poorer global satisfaction and had lower health-seeking behaviors compared to female students [9].

International students face challenges while adapting to a new country, which affect their physical and psychological health and wellbeing [10]. They consider their health challenges as minor compared to other challenges they face [9, 10]. Consequently, these students do not fully benefit from local healthcare services. Moreover, international students face barriers in accessing healthcare services in many countries, often do not sufficiently benefit from available healthcare, and delay seeking healthcare services when they are ill [11].. Delays in seeking healthcare services may result in severe health outcomes and have a potentially negative effect on public health, especially when considering infectious diseases such as COVID-19. Furthermore, fear derived from perceived stigma prevents these students from seeking health services [12]. A study conducted by Takeuchi J. and Sakagami Y. (2018) demonstrated that some international students hesitated to access mental healthcare services because of perceived stigma [13].

On the other hand, health service providers in the host countries experience challenges in providing healthcare to international students due to linguistic discordance, cultural differences, and resource constraints [14]. To ensure successful education completion, capacity growth, and productivity of international students, their health and well-being need to be monitored. While there are several studies regarding the utilization of healthcare services by migrants in Turkey, evidence of healthcare access and utilization among international students is limited. The aim of this study was to expand the current knowledge of the extent of healthcare utilization and potential barriers that affect full healthcare access among a group of international students enrolled in seven public universities in Ankara, Turkey.

## Method

### Study setting

Ankara is the capital of Turkey and is located in the northwest part of the country. It has a population of about 5.7 million people. The study population comprised international students from seven public universities in Ankara—Hacı Bayram Veli University, Ankara University, Yıldırım Beyazıt University, Gazi University, Hacettepe University, Middle East Technical University (METU) and Ankara University of Social Sciences.

### Participant recruitment and data collection

An online-based survey was conducted from the first of September to the end of October 2020. The study population consisted of international students aged 18 years and above living in Turkey for at least 1 year and enrolled in undergraduate or postgraduate programs in seven public universities in Ankara. A non-probabilistic sampling procedure was used due to logistic challenges involved in the random selection and access to some international students. Thus, the selection was non-probabilistic and by convenience sampling. However, this selection was made in a controlled basis, trying to guarantee the representability of the sample with respect to gender, nationality, university, and level of study. An online questionnaire prepared by the researchers was sent to the target group through the Student Information System (TBBS). The Computer and Information Department of the Presidency of Turks Abroad and Related Communities received the electronic questionnaire and uploaded it to the Student Information System (TBBS).

### Survey instrument

The survey instrument used was a validated semi-structured questionnaire. All information was self-reported. Sociodemographic information regarded personal attributes such as age, gender, marital status, nationality, education level, and length of stay in Turkey. Health status information included respondents’ physical activity, eating habits, smoking habits, alcohol consumption, and chronic disease. Information on healthcare services utilization included number and reasons for visiting the GP as well as access to dental, optical, mental health, student health centers, and emergency services, and in-patient admissions. Questions on the frequency of healthcare services utilization were in a “yes/no” format. If the answer was “yes”, then the respondents were asked the number of time they accessed the service.

The sociodemographic characteristics and health information were considered as independent variables, whereas utilization of healthcare services were considered as dependent variables. The variables on access to healthcare services were defined as categorical variables with two categories (i.e. no access, and one or more visits). The questions were prepared in short answer or multiple choice question type. Participants took approximately 15 min to fill the questionnaire. The purpose of the research was included on the first page of the questionnaire, informing the participants about the purpose of the study, volunteering to participate in the survey, and confidentiality of their information. After reading the first page, respondents consented to participating in the survey before filling the questionnaire.

The questionnaire was pretested on a convenience sample of 25 male and 25 female international students (*n* = 50) outside the target group to ensure clarity of interpretation and improve the validity of responses. Participants’ responses regarding the completion time, clarity of questions as well as their suggestions on improvements of the questionnaire were received, and minor changes were made. The survey instrument was validated and Cronbach’s alpha test performed to assess the internal consistency of questions.

### Statistical analysis

All statistical analyses were conducted using IBM SPSS statistics version 25. Discrete variables were presented with tables showing the number and percentage distributions. Continuous variables were described using mean, 95% CI of the mean, median, 25th and 75th percentiles, and the minimum and maximum values. Different groups within the study population were compared with cross tables. ANOVA and Chi-square tests (with Fisher’s exact test) were used to compare variables and post-hoc used to identify the source when significant differences were identified. Fisher’s exact test was used when more than 20% of the cells in the table had a frequency of less than 5. Logistic regression analysis was used to evaluate the relationships between variables related to access to health services. The confidence interval was set at 95% and *p* value of less than 0.05 accepted as statistically significant.

### Ethics statement

Ethical approval was obtained from the Non-Interventional Clinical Research Ethics Committee of Hacettepe University (No. GO 20/706), and informed consent was given by the 535 participants.

## Results

Of the total international students on Türkiye Scholarship studying in seven public universities in Ankara, 535 (28%) students from 83 countries completed the questionnaire. The sociodemographic characteristics of the participants are shown in Table 1. The mean age of the respondents was 25 years, with a minimum of 18 and a maximum of 39 years. The majority of respondents came from countries in Africa (49.2%), Middle East (24.4%), Asia (15.6%), and Europe (9.2%).

**Table 1:** Demographics of study population

Characteristic	Male(*n* = 290)		Female(*n* = 245)		Total(*n* = 535)	
	N	N%	N	N%	N	N%
Married						
Yes	48	84.1	23	9.4	69	12.9
No	244	15.9	222	90.6	466	87.1
Age Groups						
< 19	12	4.1	26	10.6	38	7.1
20–24	127	43.8	120	49.0	247	46.2
25–29	83	28.6	69	28.2	152	28.4
30–34	53	18.3	28	11.4	81	15.1
35–39	15	5.2	2	0.8	17	3.2
Living with spouse in Turkey						
Yes	22	58,5	19	82.6	41	53.9
No	31	41.5	4	17.4	35	26.1
Spouse/children Health Insurance						
Yes	11	50.0	16	84.2	27	65.9
No	11	50.0	3	15.8	14	34.1
Country of origin						
Africa	178	61.6	85	34.4	262	49.2
Middle East	70	24.2	60	24.6	130	24.4
Asia	25	8.7	58	23.8	83	15.6
Europe	13	4.2	37	15.2	49	9.2
Other regions	4	1.4	5	2.0	8	1.7
Economic status						
Good	49	16.9	70	28.6	119	22.2
Average	197	67.9	149	60.8	346	64.7
Bad	44	15.2	26	10.6	70	13.1
Level of study						
Undergraduate	133	45.9	132	53.9	265	49.5
Masters	85	29.3	66	26.9	151	28.2
Doctorate	72	24.8	47	19.2	119	22.3
Duration of stay in Turkey (years)						
1	45	15.5	51	20.8	96	17.9
2	106	36.6	91	37.1	197	36.8
3	54	18.6	46	18.8	100	18.7
4	34	11.7	23	9.4	57	10.7
5	23	7.9	19	7.8	42	7.9
> 5	28	9.7	15	6.1	21	8.0

Percentages were calculated from the number of respondents to the questions

While all the respondents received health insurance, 48.8% (*n* = 261) did not have health insurance prior to studying in Turkey. More male students had used tobacco products in their entire life compared to female students (*p* = 0.019). Of those who had used tobacco products, 12.5% (*n* = 18) smoked cigarettes daily while 36.1% (*n* = 52) had quit smoking. More female students (11.4%, *n* = 28) reported eating healthy compared to male students (*n* = 7.2%, *n* = 22). On the other hand, more male students (54.5%, *n* = 158) reported being physically active compared to female students (30.6%, *n* = 75). Of those who had a chronic disease (*n* = 14), a majority reported having chronic respiratory or cardiovascular diseases. Hospital admissions were higher among respondents with chronic diseases.

With regard to utilization of healthcare services, more female students accessed dental services compared to male students (*p* < 0.001). The bivariate analysis found that access to dental services was significantly higher among female students from European and Middle East countries and the lowest among male students from African countries (Model 2). Therapeutics, check-ups, and corrective interventions were among the most reported reasons for dentist visits.

Only 40% (*n* = 214) of the students visited Student Health Centers. The main reasons for not visiting Student Health Centers included lack of information and language barriers. Indeed, students who communicated easily when seeking health services and understood health-related instructions accessed Student Health Centers more compared to those who had language difficulties (Model 3).

Of the respondents, 31.0% (*n* = 166) reported having accessed the emergency department (ED) or taken someone they knew. The number of female students (36.3%, *n* = 89) who sort emergency services was higher compared to male students (26.6%, *n* = 77). Concerning doctor visits, no differences were obtained by gender. However, students with an extended length of stay in Turkey accessed the GP, ED, and dental services more. Of the respondents, 80% (*n* = 412) reported changing their views to access healthcare more because of the coronavirus pandemic. Three-quarter of the respondents were satisfied with the healthcare services they received.

## Discussion

There are over one hundred and eighty-five thousand international students in Turkey. International students in Turkey, like many other countries, are grappling with the reality of protecting their health during the coronavirus pandemic while away from their families. The findings of our study provide important information on access and utilization of health services by international students, and the data obtained help in providing essential health services to international students.

Findings from the study by Martin and colleagues [11] showed that the utilization of healthcare services among international students presents challenges mainly, financial and linguistic discordance. In the present study, communication barrier was reported as the main challenge for students who had difficulties explaining their complaints to healthcare providers. It was noted that economic status did not affect students’ access to healthcare services. All study participants received health insurance, and this could have potentially resulted in a lack of association between the economic level of students and healthcare access.

Previous studies have shown that international students hesitate to seek healthcare services and often delay healthcare visits when ill. Findings from our study showed that 52.0% (*n* = 278) of participants hesitated to visit the hospital when they were sick. However, three-quarters of the respondents reported that if they suspected to have infections of public health concern, such as COVID-19, they would call an ambulance. Our findings are consistent with a study by Skromanis and colleagues (2017), which demonstrated that international students regarded their health problems as minor and felt that they could manage their health challenges on their own [9]. When faced with serious health challenges, however, they prefer to seek health services.

We were also interested in analyzing the differences in international students’ health and access to health services according to gender. While there was no significant difference between male and female participants on most outcomes of interest, more male students reported eating less healthy compared to female students (Table 2). Also, more female students accessed dental and emergency health services compared to male students (Table 3). These findings support findings by Skromanis and colleagues [9] that showed male students reported poorer health status and were less likely to seek healthcare services.

**Table 2:** Lifestyle habits of the study population

Characteristic	Male(*n* = 290)		Female(*n* = 245)		Total(*n* = 535)		*P* values
	N	N%	N	N%	N	N%	
Chronic disease							
Yes	14	4.8	12	4.9	26	4.9	1.000
No	276	95.2	233	95.1	509	95.1	
Physical activity							
Active	158	54.5	75	30.6	233	43.6	0.001
Average	126	43.4	150	61.2	276	51.6	
Not active	6	2.1	20	8.2	26	4.9	
Eating habits							
Healthy	21	7.2	28	11.4	49	9.2	0.016
Average	124	43.1	124	50.6	249	46.5	
Unhealthy	144	49.7	93	38.0	237	44.3	
Tobacco use in entire life							
Yes	90	31.0	54	22.0	144	26.9	0.019
No	200	69.0	191	78.0	391	73.1	
Frequency of current smoking							
Everyday	14	15.6	4	7.4	18	12.5	0.487
Some days	12	13.3	9	16.7	21	14.6	
Rarely	31	34.4	22	40.7	53	36.8	
Do not smoke	33	36.7	19	35.2	52	36.1	
Alcohol use in entire life							
Yes	101	34.8	66	26.9	167	31.2	0.062
No	189	65.2	179	73.1	368	68.8	
Frequency of current alcohol consumption							
Everyday	18	17.8	18	27.3	36	21.6	0.241
Some days	47	46.5	33	50.0	80	47.9	
Rarely	33	32.7	13	19.7	46	27.5	
Do not drink	3	3.0	2	3.0	5	3.0	

Chi square test (with Fisher’s exact test) were used to compare variables by gender

**Table 3:** Access to healthcare facilities by study population

Characteristic	Male(*n* = 290)		Female(*n* = 245)		Total(*n* = 535)		*P* values
	N	N%	N	N%	N	N%	
Had insurance before coming to Turkey							
Yes	140	48.3	134	54.7	274	51.2	0.139
No	150	51.7	111	45.3	261	48.8	
Dentist visit							
> 1	77	26.6	96	39.2	173	32.3	0.002
No	213	73.4	149	60.8	362	67.7	
Doctor visit							
> 1	234	80.7	197	80.4	431	80.6	1.000
No	56	19.3	48	19.6	104	19.4	
Optical service access							
> 1	68	23.4	61	24.9	129	24.1	0.696
No	222	76.6	184	75.1	406	75.9	
Student health center access							
> 1	105	36.2	109	44.5	214	40.0	0.051
No	185	63.8	136	55.5	321	60.0	
Mental health service access							
Yes	5	1.7	5	2.0	10	1.9	1.000
No	285	98.3	240	98.0	525	98.1	
Emergency Services access							
Yes	77	26.6	89	36.3	166	31.0	0.010
No	213	73.4	156	63.7	369	69.0	
Hopsital admission							
Yes	32	11.0	29	11.8	61	11.4	0.771
No	258	89.0	216	88.2	474	88.6	
Communicates easily when seeking health services							
Yes	219	75.5	192	78.4	411	76.8	0.311
No	42	14.5	25	10.2	67	12.5	
Did not seek health services	29	10.0	28	11.4	57	10.7	
Inquired on what GHI covers							
Yes	84	30.2	70	29.5	154	29.9	0.943
No	194	69.8	167	70.5	361	70.1	
Coronavirus pandemic changed views on health access							
Yes	214	77.0	198	83.5	412	80.0	0.063
No	64	23.0	39	16.5	103	20.0	
Hesitate to visit hospital when ill							
Yes	153	52.8	125	51.0	278	52.0	0.114
No	132	45.5	108	44.1	240	44.9	
Unsure	5	1.7	12	4.9	17	3.2	

Chi square test (with Fisher’s exact test) was used to compare variables by gender

*GHI* General health insurance

Studies by Augustinos and colleagues (2011) showed that a lack of knowledge of health system organization and cultural differences presented challenges to full healthcare access and utilization by international students [8]. Findings from our study showed that students who had lived in Turkey for more than 5 years accessed ED, GP, and dental services more (Table 4). Students with a longer stay adapt to the social, culture, and language of the host countries. They have social support networks and understand the organization of the local health system. Therefore, these students access and utilize health services more compared to students who have a shorter stay.

**Table 4:** Logistic regression of variables potentially associated with health access of study population

	Model 1 Hospital Admission		Model 2 Access to Dentist		Model 3 Access to SHC		Model 4 Access to ED		Model 5 Access to GP	
	χ2 = 72.48, *p* < 0.001 (*n* = 535)		χ2= =100.73, *p* < 0.001 (*n* = 535)		χ2 = 84.24, *p* < 0.0001 (*n* = 535)		χ2 = 70.49, *p* < 0.001 (*n* = 535)		χ2 = 155.98, *p* < 0.001 (*n* = 535)	
	OR	95% CI	OR	95% CI	OR	95% CI	OR	95% CI	OR	95% CI
Gender										
Male	1.00		1.00		1.00		1.00		1.00	
Female	1.62	0.85–5.10	2.04	1.28–3.26*	1.41	0.93–2.16	1.59	1.02–2.48*	1.15	0.62–2.14
Age, continous	1.20	1.05–1.37*	1.06	0.96–1,17	1.00	0.92–1.11	0.97	0.88–1.05	1.14	1.00–1.29*
Country										
Africa	1.00		1.00		1.00		1.00		1.00	
Asia	1.37	0.57–3.31	1.35	0.72–2.52	1.05	0.58–1.88	1.54	0.84–2.84	0.89	0.37–2.04
Europe	0.38	0.08–1.87	2.419	1.16–5.06*	1.03	0.52–2.06	1.39	0.66–2.92	0.79	0.30–2.10
Middle East	1.39	0.67–2.88	2.056	1.23–3.44*	0.78	0.47–1.27	2.45	1.49–4.07*	0.77	0.38–1.54
Other regions	3.67	0.65–2.05	1.51	0.32–7.19	1.12	0.25–5.00	1.62	0.36–7.41	0.74	0.10–5.64
Chronic disease										
No	1.00				1.00		1.00		1.00	
Yes	8.28	2.83–24.22*	–	–	1.46	0.60–3.60	2.56	1.01–6.49*	3.41	.65–8.04
Duration of stay										
1	1.00		1.00		1.00		1.00		1.00	
2	3.03	1.02–9.03*	1.74	0.89–3.40	1.25	0.66–2.40	2.08	1.09–3.96*	4.48	2.20–9.05*
3	1.95	0.60–6.36	2.74	1.31–5.73*	1.62	0.75–3.71	2.08	1.09–4.32*	4.28	1.18–10.09*
4	2.23	0.62–8.09	3.88	1.67–9.02*	1.00	0.44–2.30	2.10	0.88–5.00	9.08	2.28–36.15*
5	2.16	0.53–8.89	4.38	1.79–10.76*	0.37	0.15–0.93*	3.11	1.28–7.60*	3.17	1.06–9.45*
Ease of Communiation										
No	1.00		1.00		1.00		1.00		1.00	
Yes	.93	0.38–2.20	0.55	0.3–1.05	2.37	1.28–4.37*	0.58	0.32–1.04	1.71	0.78–3.74

*SHC* student health center, *ED* emergency department, *GP* general practitioner

*indicate P < 0.05 on comparison with the reference category

### Implications of study

Barriers to healthcare access and utilization reported by international students in the current study, which need to be addressed in healthcare delivery programs and health promotion, include lack of awareness of health support systems, perceived stigma associated with mental healthcare services, and language barrier. In addition to the stress associated with the transition to university, international students often face stress associated with adapting to the local language, social, and cultural way of life. The mental wellbeing of international students is as important as their physical health. Participants in the study (22.6%, *n* = 121) reported fear of seeking mental healthcare because they were foreigners. Perceived stigma hinders access to essential health services and this affects the learning and wellbeing of international students.

To further support existing programs to improve the accessibility of health service delivery to international students, culturally specific health promotion programs reinforced consistently during the academic year could be introduced. International students experience less sense of community in general, which affects their well-being. Education providers have an essential role in monitoring the health and well-being of international students.

### Strengths and limitations of the study

The strength of this work is in the diversity of the study population. Participants from eighty-three countries were enrolled in the study. In terms of the country of birth and age, the study population was representative of international students in Turkey. The study also managed to recruit a relatively large sample of both male and female participants. On the other hand, international students paying for their education expenses were not included in the study due to logistic challenges in data collection. The findings of this study provide information on healthcare access and utilization among international students in Turkey on scholarship who receive healthcare insurance. To ensure that there was no potential systematic differences between respondents and non-respondent, we verified that the respondents were a representative of the target population with respect to university, nationality, level of study, and gender.

## Conclusion

The findings of our study not only contribute to the existing literature on health utilization but also reveal several challenges faced by international students in Turkey. This study offers policymakers and hospital administrators with insight into the experiences faced by international students most of whom may not have a direct avenue to give feedback to key decision-makers. According to the survey results, the lack of awareness, language barrier, and fear caused by perceived stigma hinder these students from fully accessing health services. This may be disastrous in case a student with an infectious disease does not seek medical attention and ends up infecting other people. It can also be catastrophic on the patient if at all the disease could have been treatable during the early stages but they end up not seeking treatment. Therefore, the data obtained show the need for and will inform the provision of targeted health services to international students in Turkey.

In particular, it calls for more awareness campaigns among international students on the how, when, where, and from whom certain health-related services can be obtained. Such support can be provided by a designated officer in the international students office. Secondly, language barrier can be improved by having important information, documents as well as websites having alternative languages for international students to use. This is important in helping to avoid misinformation and miscommunication. Hospital emergency and information call centers can also benefit from optional languages that the callers can make in case of inquiry or emergency. That said, the efforts made by the Turkish government to include international students, especially those benefiting from the government-sponsored scholarships, into its national health insurance scheme is commendable and allows thousands of students improved access to healthcare.

## Data Availability

The datasets supporting the conclusions of this article are available from the corresponding author, and can be shared upon reasonable request.
